# Oxygen Dynamics in the Rhizosphere of *Vallisneria spiralis* Characterized by a Fluorescent Planar Optode

**DOI:** 10.3390/plants15131935

**Published:** 2026-06-23

**Authors:** Jingwei Tan, Zhihao Wu, Xiaosong Yang, Weidong Jin, Yiming Zhao, Qing Cai

**Affiliations:** 1 National Engineering Laboratory for Lake Pollution Control and Ecological Restoration, Institute of Lake Environment, Chinese Research Academy of Environmental Sciences (CRAES), Beijing 100012, Chinawu_zhihao1@163.com (Z.W.);; 2 State Environmental Protection Key Laboratory for Lake Pollution Control, Institute of Lake Environment, Chinese Research Academy of Environmental Sciences (CRAES), Beijing 100012, China; 3 State Key Laboratory of Environmental Criteria and Risk Assessment, Chinese Research Academy of Environmental Sciences (CRAES), Beijing 100012, China

**Keywords:** rhizosphere, *Vallisneria spiralis*, planar optode, two-dimensional, radial oxygen loss, photosynthesis

## Abstract

Oxygen (O_2_) leakage in macrophyte rhizospheres is an adaptive strategy for hypoxic environments, which is important in lake ecological restoration. In this investigation, the fluorescent planar optode (PO) technique is used for two-dimensional (2D) distribution of dissolved O_2_ at a submillimeter scale in the rhizosphere of *Vallisneria spiralis* under various environmental conditions. The spatial heterogeneity in the distribution of oxic microniches is frequently verified in the rhizosphere. The radial oxygen loss (ROL) rate for root systems is characterized by the following sequence: basal root (20.6 ± 5.1–49.6 ± 9.5 nmol m^−2^ s^−1^, n = 7) > lateral root (14.1 ± 4.1–36.6 ± 8.3 nmol m^−2^ s^−1^, n = 7) > root tip (13.1 ± 4.6–28.8 ± 6.4 nmol m^−2^ s^−1^, n = 7). The O_2_ maximum value on lines transecting each kind of root also obeys the sequence mentioned above. For one typical root, (1) O_2_ decreases from 131.2 ± 2.4–147.4 ± 3.7 μmol L^−1^ at the root center to 47.2 ± 1.4–75.9 ± 2.2 μmol L^−1^ in the rhizosphere fringe due to O_2_ supply from the root surface and O_2_ consumption in rhizosphere sediment, and (2) the furthest distance from the aboveground part to the root tip leads to the lowest O_2_ concentration at the root apex of that root. The light/dark transition and O_2_ level in overlying water modulate the photosynthetic activity of leaves and the transfer of oxygen in the water column through aerenchyma tissue to the roots. The sequence of the oxygenated area (%), ROL rate, and O_2_ concentration in rhizosphere sediment under various conditions is demonstrated as: high illumination/high O_2_ > darkness/high O_2_ > high illumination/low O_2_ > darkness/low O_2_. The effect of O_2_ in water on the ROL of *Vallisneria spiralis* is more distinct than illumination. Oxygen storage in roots, and especially O_2_ diffusion from overlying water, can supplement O_2_ deficiency in the rhizosphere during the cessation of photosynthesis under darkness. This research advances the understanding of complex interrelationships among O_2_ dynamics in different root parts, photosynthesis, O_2_ in overlying water and O_2_ transfer through plant aerenchyma to the rhizosphere.

## 1. Introduction

Oxygen (O_2_) can be transported to the rhizosphere of aquatic macrophytes or terrestrial plants through aerenchyma tissue [1,2,3,4]. Radial oxygen loss (ROL) in the macrophyte rhizosphere is mainly controlled by photosynthesis and O_2_ consumption through the root system [5]. Photosynthetic O_2_ production under light exposure controls partial O_2_ pressure in the internal gas channel of the plant, leading to root O_2_ leakage [6]. O_2_ in overlying water maintains aerobic conditions around the roots through O_2_ diffusion from overlying water when there is no O_2_ supply through leaf photosynthesis during the night [7]. O_2_ release in the rhizosphere can engender the formation of a spatiotemporal microoxic zone and iron (Fe) plaque on the root surface [4]. ROL can affect biogeochemical reactions in rhizosphere sediment, such as nitrification, metal mobilization, and contaminant degradation. O_2_ secretion in the rhizosphere can also protect roots from the penetration of toxins [8,9,10].

O_2_ leakage patterns in root systems are substantially different among plant species. Some aquatic plants (*Zostera marina*, *Halophila ovalis* and *Phragmites australis*) mainly secrete O_2_ through the root tip [11,12,13]. Meanwhile, O_2_ can be released from all root parts (basal root, lateral root and root tips) of *Potamogeton crispus*, *Cymodocea rotundata*, *Myriophyllum spicatum*, *Potamogeton perfoliatus* and *Vallisneria spiralis* (*V. spiralis*) [5,14,15,16,17]. O_2_ depletion from the root center to the rhizosphere edge in the cross-section of a typical plant root is due to O_2_ leakage from the root surface and O_2_ consumption in the rhizosphere and bulk sediments [18].

Conventional techniques for the measurement of O_2_ concentration in the macrophyte rhizosphere mainly include the use of needle-type microelectrodes [19,20], optical fibers [21], and the colorimetrical method [22]. However, these techniques have some inherent drawbacks, including low accuracy, complex operation, the large sample volume required, electrode brittleness, extensive replication to cover large areas, and incompatibility with two-dimensional (2D) analysis. Thus, an in situ and non-destructive technique—the planar optode (PO)—has been developed for the 2D distribution of O_2_ in the rhizosphere of aquatic macrophytes. The PO system for O_2_ measurement consists of an O_2_-sensing film (luminophore immobilized in one analyte-permeable matrix coated onto a supporting material), an excitation unit, and an imaging system (camera) [23,24,25,26]. PO measurement is based on dynamic quenching of photoluminescence by O_2_ under light excitation without O_2_ consumption [22,27,28,29]. The advantages of PO measurement include cheapness, high spatial coverage (mm^2^–cm^2^), high spatial resolution (-μm), measurement period flexibility (seconds–days), and high selectivity and sensitivity [30].

The PO technique has been used to determine the O_2_ dynamics of biological samples [31], coral [32], sediment [33], biofilms [30], animal burrows [34] and plant–root systems [12,35,36]. The measurement of the 2D distribution of O_2_ in the plant rhizosphere using POs has become a research hotspot in recent years [12,35,36], and these plants include (1) aquatic macrophytes such as *Phragmites australis*, *V. spiralis* or *Potamogeton crispus* and (2) terrestrial plants such as rice plant and *Elymus athericus* [4,6,13,15,37,38,39]. The root-triggered redox condition influences speciation and biogeochemical reactions of nitrogen, phosphorus or trace metals in rhizosphere sediment [38,40,41].

*V. spiralis* is usually used for lake ecological restoration due to its high capability of O_2_ leakage from roots, tolerance of trace metals and nutrients and bioaccumulation [36]. Understanding ROL patterns in various root parts of *V. spiralis* under different environmental conditions is important for elucidating its resilience and adaptability to a fluctuating environment and phytoremediation mechanisms for lake ecology.

Until now, only a few papers have reported O_2_ dynamics in the rhizosphere of *V. spiralis* using the PO technique [6,10,36], and the characteristics of O_2_ distribution in the root zone and the driving mechanisms for ROL have not been revealed clearly. Two questions are proposed, including: (1) Do ROL parameters and O_2_ concentrations vary among different locations in the rhizosphere (basal root, lateral root, root tip or some lines across or along one typical root)? (2) What are the influences of the light/dark transition and O_2_ levels in overlying water on ROL in various root parts and the driving mechanisms? In order to answer those questions, the PO technique is used for the high-resolution quantitative characterization of the 2D O_2_ distribution in the rhizosphere of *V. spiralis* in this investigation. The research results should improve our understanding of O_2_ cycling at the root–sediment interface related to lake ecological remediation using aquatic macrophytes.

## 2. Materials and Methods

### 2.1. Sediment Sampling and Plant Pre-Incubation

**Figure 1:**
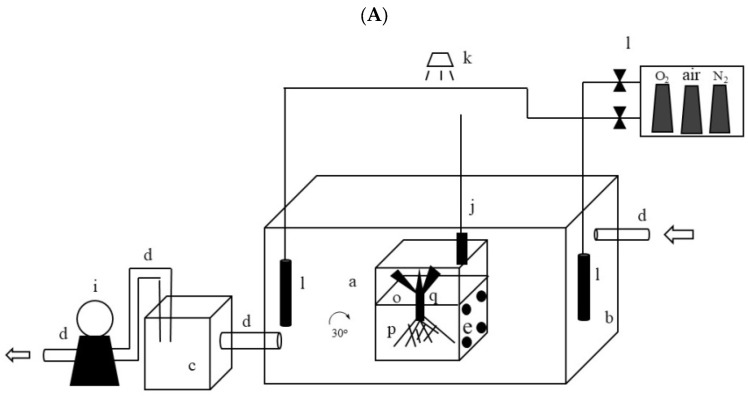

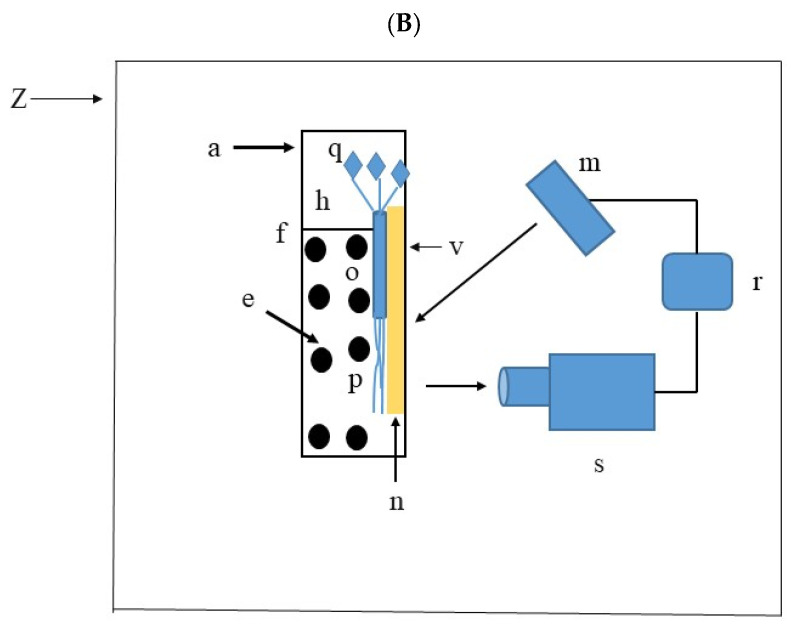
Schematic graphs of the experimental design. (**A**): The overall schematic graph for the experiment. (**B**): The schematic graph of the rhizobox and the planar optode (PO) instrument. (a) Rhizobox; (b) experimental flume; (c) water return tank; (d) return pipe; (e) sediment; (f) sediment/water interface; (h) water; (i) peristaltic pump; (j) O_2_/T/pH electrode; (k) illumination lamp; (l) aeration device; (m) light-emitting diode (LED); (n) O_2_-sensing film; (o) aquatic macrophyte; (p) root; (q) leaf; (v) detachable front window; (r) trigger box; (s) camera; (z) enclosed PO device.

In April 2023, sediment (thickness: 10 cm; weight: 20 kg) and overlying water (50 L) were collected using an Ekman grab sampler and an acid-cleaned polyethylene bottle, respectively, from one site in the Qing River in Beijing (China) (Figure S1, Supplementary Materials). The macrofauna and debris were removed from the sediment. The sediment was air-dried and ground to pass through a screen with a 100 µm mesh size. The sediment was mixed and homogenized. The total phosphorus (TP), total nitrogen (TN) and total iron (TFe) in the sediment were 984, 2889 and 15,854 mg kg^−1^ (dry weight), respectively. Seedlings of *V. spiralis* were purchased from Runxi Agricultural Science and Technology Co., Ltd. (Xiong’an New Area, Henbei Province, China). First, 200 mL of water and the dry sediment (0.8 kg) were mixed and put into a rhizobox (Figure 1A). The *V. spiralis* seedlings (length: 10 ± 0.4 cm; weight: 3.2 ± 0.2 g) were planted into the sediment in the rhizobox. The rhizobox was put into one experimental flume (Figure 1A) for 12 d of cultivation before PO measurement. The rhizobox was placed at an angle of 30° in the experimental flume to ensure root development along the front window of the rhizobox (Figure 1A). *V. spiralis* was cultivated at 20 °C under artificial irradiance with wave length range (350–1100 nm) by a light-emitting diode (LED) (Figure 1A) with a daily cycle of 12 h light (irradiation intensity: 150 μmol photons m^−2^ s^−1^)/12 h dark (irradiation intensity: 0 μmol photons m^−2^ s^−1^) and O_2_ (175 μmol L^−1^) in water in the experimental flume. The irradiation intensity was measured using a light quantum meter (3415 FSE, Spectrum Technologies, Inc., Aurora, IL, USA). The detailed plant cultivation is described in the Supplementary Materials (SM A).

### 2.2. Experimental Setup

After 12 d of cultivation, the rhizobox was taken out from the experimental flume, and the detachable front window was removed from the rhizobox (Figure 1). The other detachable front window with a square O_2_-sensing film (8 cm × 8 cm, EasySensor) adhered to it (Figure 1) was immediately fixed in the rhizobox. Black plastic sheeting was used to cover the outside of the rhizobox to keep the rhizosphere dark. The rhizobox was put back in water in the experimental flume and maintained for 4 h under one environmental condition. Four environmental conditions were regulated by aeration equipment and an illumination lamp (Figure 1A), including illumination (200 μmol photons m^−2^ s^−1^)/O_2_ (141 μmol L^−1^), darkness (0 μmol photons m^−2^ s^−1^)/O_2_ (141 μmol L^−1^), illumination (200 μmol photons m^−2^ s^−1^)/O_2_ (280 μmol L^−1^) and darkness (0 μmol photons m^−2^ s^−1^)/O_2_ (280 μmol L^−1^). These conditions are designated as high illumination/low O_2_, darkness/low O_2_, high illumination/high O_2_ and darkness/high O_2_. A multi-parameter water quality analyzer (HQ 4300, Hach Company, Loveland, CO, USA) (Figure 1) was used to measure O_2_, temperature and pH in the water in the rhizobox. The temperature and pH were 20 °C and 8.0, respectively. The irradiation intensity was measured using a light quantum meter (3415 FSE, Spectrum Technologies, Inc., Aurora, IL, USA). This light quantum meter specifically provides accurate illumination readings of PAR (photosynthetically active radiation) with a wavelength range (400–700 nm) (Figure 1). 

After 4 h of cultivation, the rhizobox was retrieved from water, and the outside black plastic sheeting was removed. It was transferred to the enclosed PO device (Figure 1B) for O_2_ measurement. Then, the rhizobox was put back into the experimental flume for the next 4 h of cultivation. The disturbance of O_2_ in the rhizosphere sediment caused by changing the detachable front window in the rhizobox or measuring rhizosphere O_2_ in the PO device mentioned above can be ignored after 1 h of cultivation in water. After PO measurement for O_2_ in rhizosphere sediment under all four environmental conditions, the detachable front window of the rhizobox was removed, and a digital camera (Canon EOS 600D, Canon Inc., Tokyo, Japan) was used to take photos of the rhizosphere. The diameters of the basal root, lateral root and root tip were measured using a digital vernier caliper (543-781, Mitutoyo Company, Takatsu-ku, Kawasaki, Kanagawa, Japan). The cultivation of *V. spiralis* and the procedures for roots and sediment after PO measurement are described in SM A (Supplementary Materials).

### 2.3. PO Imaging

PO measurement of oxygen is based on the dynamic quenching of a fluorophore in the presence of O_2_. The O_2_-sensing film with a thickness of 10 µm was made by EasySensor Ltd. (Nanjing, China) based on the method recommended in references [22,36,42,43]. First, a sensor “cocktail” is prepared by dissolving an oxygen-quenchable luminophore, i.e., a reference fluorophore coumarin C545 (C545T), Platinum (II)5,10,15,20-tetrakis-(2,3,4,5,6-pentafluorphenyl) porphyrin (PtTFPP) and polystyrene in toluene. This “cocktail” is gently stirred and then sprayed on a transparent dust-free polyethylene terephthalate using ultrasonic spray equipment [22,36]. Second, one thin layer of silicone rubber (Dow Corning 3140, Dow Corning Corporation, Midland, MI, USA) is coated on top of this dry sensing membrane as a protective layer [36]. Detailed information on the O_2_-sensing film mentioned above can be found in SM B.

The PO device (Easysensor PO 2100) (Figure 1B) was purchased from EasySensor Ltd. (Nanjing, China). The PO device contains a Complementary Metal Oxide Semiconductor (CMOS) camera with a 460 nm long-pass filter, two light-emitting diodes (LEDs) (390–400 nm), a PC-controlled trigger box for the camera and LEDs, and a darkroom. The recorded fluorescence image with a 2D spatial resolution of 62 × 62 µm is calibrated using the fluorescence intensities of the known O_2_ concentrations (0% and 100% O_2_ saturation in water) at 20 °C, using a modified Stern–Volmer equation (Equation (1)) [44].

(1) $$\frac{R}{R_{0}}=\alpha +(1-\alpha )\frac{1}{1+K_{SV}C}$$

where *R* and *R*_0_ are the fluorescence intensity ratio (red/green) at an O_2_ concentration of *C* (%, air saturation) and *C*_0_ (0%, air saturation), respectively. *C* is the O_2_ concentration, *K_sv_* is the Stern–Volmer quenching constant, and α is the non-quenchable fraction of the fluorescence signal, which is temperature independent. Details of the calibration and calculation of the O_2_ optode are provided in SM B.

### 2.4. Data Analysis

The fluorescent image was processed using Image J. 1.53 (http://rsb.info.nih.gov/ij/, accessed on 2 March 2023) and divided into red, green and blue channels. The red/green ratio at each point in the fluorescent image was converted to O_2_ concentration by calibration (SM B). The detection limit of PO analysis (2.20 μmol L^−1^) is quantified as three times the standard deviation (3SD) of the measured concentration in an anoxic area of the fluorescent image (about 1% air saturation; n = 7). The point where the O_2_ concentration drops below 2.20 μmol L^−1^ is considered to be the O_2_ penetration depth (OPD) (mm) across the sediment/water interface (SWI) and the root/sediment interface. The diffusive O_2_ uptake (DOU) (nmol m^−2^ s^−1^) is derived according to the vertical O_2_ concentration profile at the SWI and Fick’s first law of diffusion (Equation (2)) [13]:

(2) $$\mathrm{DOU}\;=\;\phi \;\times \;Ds\;\times \;\frac{\partial C}{\partial z}$$

where *ϕ* represents the sediment porosity derived using the method (SM A) proposed in reference [45]; *ϕ* equals 0.81 ± 0.03 (n = 7) for the rhizosphere sediment in this experiment; Ds (cm^2^/s) is the molecular diffusive coefficient of O_2_ in sediment, and it can be calculated by Equation (3) [46]:


(3) $$D_{s}\;=\;\phi^{2}D_{0}$$

D_0_ is the oxygen diffusive coefficient in water, and *∂C/∂z* is the O_2_ concentration gradient. In this investigation, seven O_2_ vertical profiles in each 2D image of the O_2_ distribution (for example, Figure S2) were chosen for derivation of $\frac{\partial C}{\partial Z}$. The average volume-specific O_2_ consumption (R_SWI_) (μmol m^−3^ s^−1^) is calculated through Equation (4) [13]: 


(4) $$R_{SWI}\;=\;DOU/OPD$$

The ROL rate (nmol m^−2^ s^−1^) from the roots of *V. spiralis* can be calculated using Equation (5):

(5) $$ROL\;rate\;=\;\phi \;\times \;R_{SWI}\;\times \;L\;\times \;(L/2A\;+\;1)$$

where L (mm) is half the width of the oxygenated zone and A (mm) is the average root diameter [13,47].

**Figure 2:**
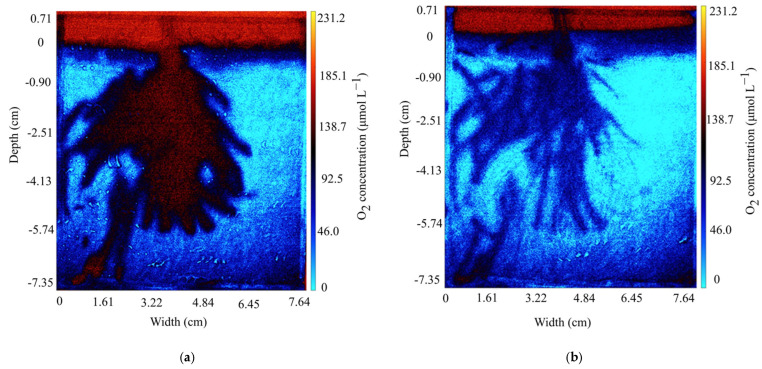

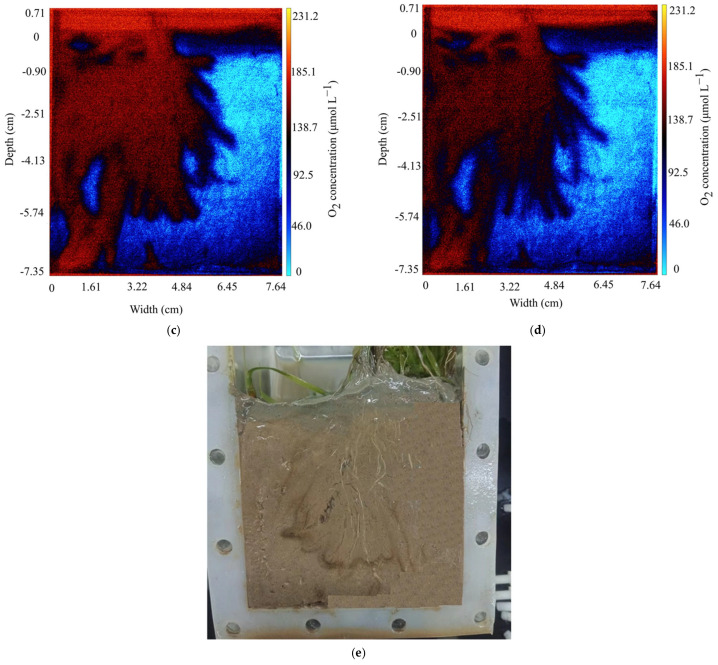
Images of the 2D distribution of O_2_ in rhizosphere sediment under four environmental conditions of (**a**): high illumination/low O_2_; (**b**): darkness/low O_2_; (**c**): high illumination/high O_2_ and (**d**): darkness/high O_2_. (**e**): An image of roots and rhizosphere sediment. Notice: The “blue” and “red” colors in the color bars and subfigures (**a**–**d**) reflect the change in O_2_ concentration from low to high levels.

Images of the 2D O_2_ distribution in the rhizosphere were drawn using Surfer software (Version 11.0, Golden Software, LLC, Golden, CO, USA). All O_2_ concentration data in vertical O_2_ profiles or 2D distribution images and ROL parameters are presented as “mean ± standard deviation (SD), n = 7”. Statistical analyses were performed using SPSS software (Version 26.0, IBM Corporation, Armonk, NY, USA). The normality of data distributions was examined via the one-sample Kolmogorov–Smirnov test, and homogeneity of variance was assessed using Levene’s test. All oxygen concentrations and ROL datasets conformed to a normal distribution and homoscedasticity (*p* > 0.05). One-way analysis of variance (one-way ANOVA) was used for comparisons among multiple data groups, with the significance level set at *p* < 0.05. Seven vertical oxygen profiles (Figure S2) for data analysis were selected from each image (Figure 2) at an interval of 0.70 cm. Each vertical profile contained 1300 data points.

## 3. Results

### 3.1. The Parameters for O_2_ Dynamics in Rhizosphere Sediment

The O_2_ concentration distributions in rhizosphere sediment under four environmental conditions are demonstrated in Figure 2. Differences in O_2_ concentrations among (1) datasets in seven vertical profiles (Figure S2) in each sub-image (Figure 2) or (2) those in four subfigures (Figure 2) under the four environmental conditions were all statistically significant, as evaluated by one-way ANOVA (*p* < 0.05). The parameters for O_2_ leakage in the rhizosphere are presented as “mean ± SD (n = 7)”, as demonstrated in Table 1. OPD and DOU just below the SWI varied by 1.30 ± 0.20–11.14 ± 2.24 mm (n = 7) and 10.8 ± 1.6–14.3 ± 3.0 nmol m^−2^ s^−1^ (n = 7) under the four environmental conditions, respectively. *R_SWI_* in the sediment ranged from 1.2 ± 0.2 to 1.8 ± 0.4 µmol m^−3^ s^−1^ (n = 7). ROL rates for three root parts under various environmental conditions followed the sequence: basal root > lateral root > root tip. Regarding environmental conditions, the ROL rate followed the sequence: high illumination/high O_2_ > darkness/high O_2_ > high illumination/low O_2_ > darkness/low O_2_ (Table 1).

**Figure 3:**
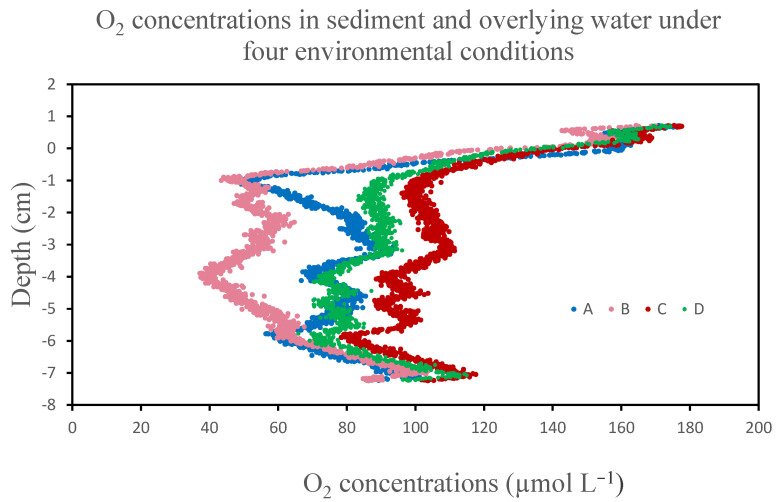
The vertical O_2_ concentration profiles derived from 2D images of the O_2_ distribution under four environmental conditions. A: high illumination/low O_2_; B: darkness/low O_2_; C: high illumination/high O_2_ and D: darkness/high O_2_.

The vertical O_2_ profiles derived from 2D images of the O_2_ distribution in rhizosphere sediment and overlying water under the four environmental conditions are demonstrated in Figure 3. The sequence of the average O_2_ concentration in rhizosphere sediment under the four environmental conditions was: high illumination/high O_2_ (101.7 ± 2.3 μmol L^−1^, n = 7) > darkness/high O_2_ (88.2 ± 1.6 μmol L^−1^, n = 7) > high illumination/low O_2_ (79.8 ± 1.2 μmol L^−1^, n = 7) > darkness/low O_2_ (62.5 ± 1.2 μmol L^−1^, n = 7). There were a lot of roots located at the surface rhizosphere sediment layer (depth: 0–−5 cm) (Figure 2e). The average O_2_ (57.2 ± 1.0 µmol L^−1^, n = 7) in surface rhizosphere sediment under darkness/low O_2_ was significantly lower than 81.4 ± 1.2–104.1 ± 1.9 µmol L^−1^ (n = 7) under the other three conditions with high illumination and (or) O_2_ in water (Figure 3) (*p* < 0.05). ROL rates (28.8 ± 6.4–49.6 ± 9.5 nmol m^−2^ s^−1^, n = 7) under high illumination/high O_2_ (Table 1) were significantly higher than 13.1 ± 4.6–39.2 ± 7.5 nmol m^−2^ s^−1^ (n = 7) under the other three conditions (*p* < 0.05). Furthermore, ROL rates under high illumination/high O_2_ also corresponded to the largest O_2_ peaks (111.4 ± 2.4 μmol L^−1^ at −3.196 cm, 103.7 ± 2.8 μmol L^−1^ at −4.523 cm, 101.6 ± 2.2 μmol L^−1^ at −5.354 cm, 117.6 ± 3.2 μmol L^−1^ at −7.052 cm, n = 7) in the vertical O_2_ profile (Figure 3) of the four environmental conditions.

### 3.2. O_2_ Dynamics in Rhizosphere Sediment and Around Different Root Parts

**Figure 4:**
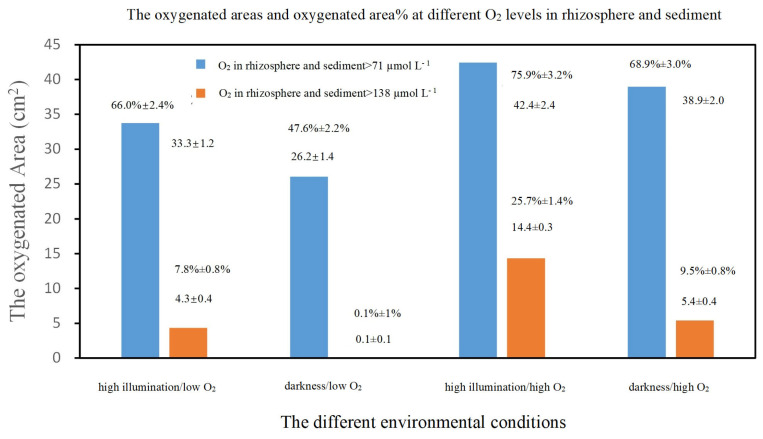
The oxygenated area (cm^2^) for O_2_ > 71 μmol L^−1^ (condition *α*) or >138 μmol L^−1^ (condition *β*) and the percent of those areas in the whole rhizosphere sediment zone (oxygenated area %) under four environmental conditions of high illumination/low O_2_, darkness/low O_2_, high illumination/high O_2_ and darkness/high O_2_.

The oxygenated area (cm^2^) for O_2_ > 71 μmol L^−1^ (condition *α*) or >138 μmol L^−1^ (condition *β*) and the percent of the oxygenated area in the whole rhizosphere sediment (area %) under various environmental conditions were derived using Image. J. 1.53 software, as demonstrated in Figure 4. The largest area % values of 75.9% ± 3.2% (n = 7) for condition *α* and 25.7% ± 1.4% (n = 7) for condition *β* under high illumination/high O_2_ of the four environmental conditions (Figure 4) corresponded to the highest ROL rate (28.8 ± 6.4–49.6 ± 9.5 nmol m^−2^ s^−1^) (Table 1) and the average O_2_ concentration (101.7 ± 2.3 µmol L^−1^) in the rhizosphere sediment (Figure 3).

**Figure 5:**
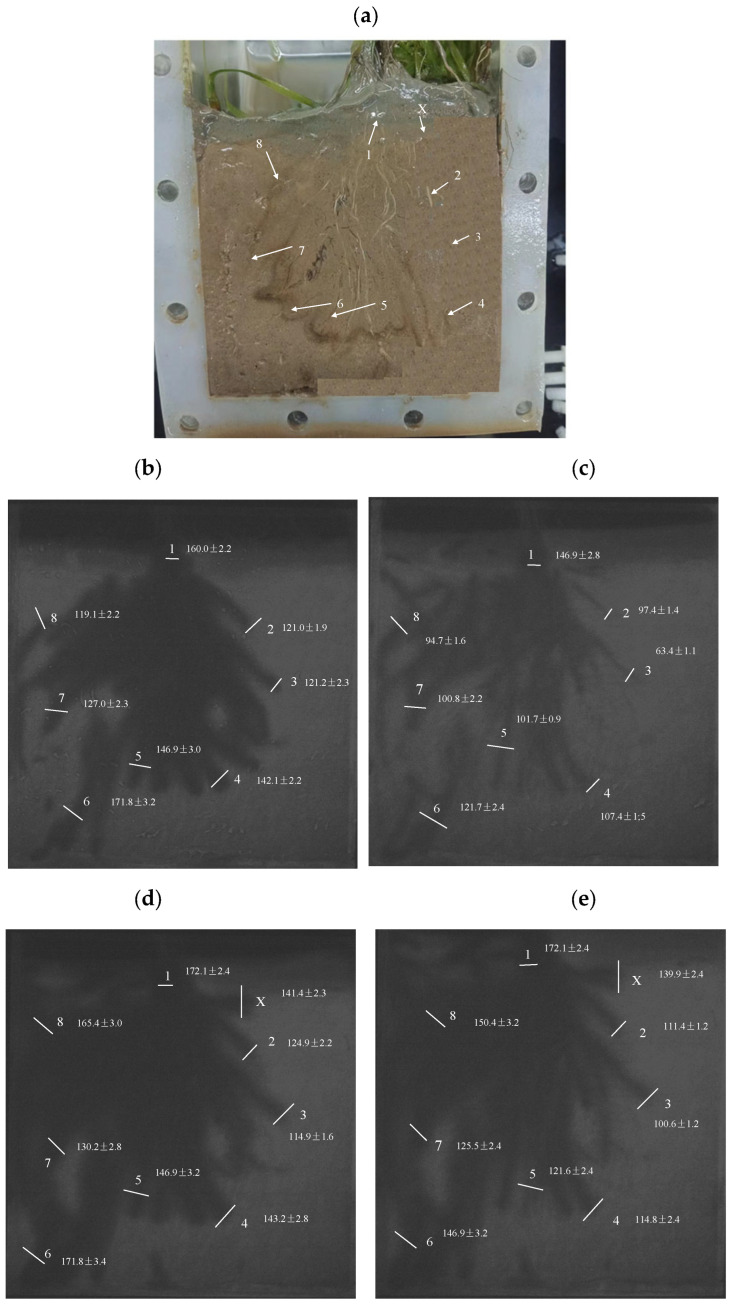
(**a**): The locations of nine roots in the root system of *V. spiralis* in a photograph. The locations of the line transecting the root tip, lateral root and basal root and the related maximum O_2_ concentrations in 2D fluorescent images under four environmental conditions, including: (**b**): high illumination/low O_2_, (**c**): darkness/low O_2_, (**d**): high illumination/high O_2_ and (**e**): darkness/high O_2_.

The maximum O_2_ concentrations for the lines transecting the basal root (1), root tip (2, 3, 4 or X) and lateral root (5, 6, 7 or 8) under the four conditions are demonstrated in Table 2. Images of the rhizosphere sediment, the locations of different roots and the related maximum O_2_ concentrations in PO images under the four environmental conditions are demonstrated in Figure 5. The basal root (1) is the central root of *V. spiralis*, and it spreads downward in sediment (Figure 5a); the root tip (2, 3, 4 or X) on the right of Figure 5a is defined as a root segment with a distance of 0–1.5 mm from the root spire; the lateral root (5, 6, 7 or 8) on the left of Figure 5a is a branch root growing out of a base root. The distinct O_2_ leakage from the root tip (X) can only be found in Figure 5d (high illumination/high O_2_) and Figure 5e (darkness/high O_2_).

For the basal root (1), the maximum O_2_ concentration of 172.1 ± 2.4 µmol L^−1^ (n = 7) (root line 1) under high illumination/high O_2_ or darkness/high O_2_ was larger than 160.0 ± 2.2 µmol L^−1^ (n = 7) under high illumination/low O_2_ and 146.9 ± 2.8 µmol L^−1^ (n = 7) under darkness/low O_2_. In sum, the average O_2_ maximum value for different root types under the four conditions (Table 2) followed the sequence: basal root > lateral root > root tip. The O_2_ maximum in the basal root under the four conditions (Table 2) followed the sequence: high illumination/high O_2_ = darkness/high O_2_ > high illumination/low O_2_ > darkness/low O_2_. For the root tip and the lateral root, the average O_2_ maximum under the four conditions (Table 2) followed the sequence: high illumination/high O_2_ > high illumination/low O_2_ > darkness/high O_2_ > darkness/low O_2_.

**Figure 6:**
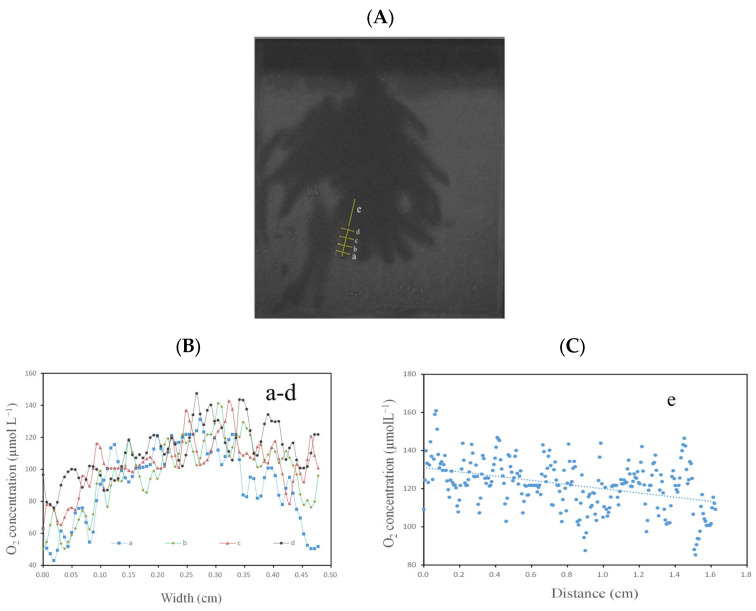
The cross-sectional profiles (a–d) for O_2_ distribution across one typical root and the longitudinal line (e) for O_2_ distribution along the root axis under high illumination/low O_2_. (**A**): A fluorescent image of O_2_ with one root and five lines (a–e); (**B**): O_2_ distribution across the cross-sectional profiles (a–d) across the root; (**C**): O_2_ distribution for the longitudinal line (e) along the root axis.

Four radial O_2_ lines (a–d) transect across the different locations of a typical root surface, and line (e) is the longitudinal O_2_ profile along this root axis, as demonstrated in Figure 6A. This root is extracted from root 5 under high illumination/low O_2_ in Figure 5b. For the four lines (a–d) across the root, the maximum O_2_ concentrations of 131.2 ± 2.4 μmol L^−1^ (n = 7) (line a), 141.2 ± 2.8 μmol L^−1^ (n = 7) (line b), 136.8 ± 3.2 μmol L^−1^ (n = 7) (line c) and 147.4 ± 3.7 μmol L^−1^ (n = 7) (line d) appeared in the root center; after that, they gradually dropped to the lowest values of 47.2 ± 1.4 μmol L^−1^ (line a), 50.3 ± 1.6 μmol L^−1^ (line b), 63.0 ± 2.0 μmol L^−1^ (line c) and 75.9 ± 2.2 μmol L^−1^ (line d) (n = 7) at the rhizosphere fringe (Figure 6B). The O_2_ concentration distribution of the longitudinal O_2_ profile (e) along the root axis with a length of 1.62 cm is demonstrated in Figure 6C. O_2_ concentrations gradually decreased from a maximum of 160.9 ± 3.2 μmol L^−1^ (n = 7) at the top of the root axis to a minimum of 85.3 ± 2.4 μmol L^−1^ (n = 7) near the root tip. Altogether, (1) the sequence of the average O_2_ concentrations in the basal root, lateral root and root tip under the four environmental conditions and (2) the sequence of O_2_ concentrations in different locations on lines (a–e) in one typical root under high illumination/low O_2_ are demonstrated in Table 3.

## 4. Discussion

### 4.1. O_2_ Leakage Patterns and Oxidation Expansion in the Rhizosphere Characterized by ROL Parameters

O_2_ leakage from *V. spiralis* led to conspicuous oxygenated zones around roots (Figure 2) [6,37]. O_2_ concentrations were depleted significantly from the maximum in the root surface to the minimum in the rhizosphere fringe and bulk sediment (Figure 2 and Figure 5), and the reflected O_2_ decrease was controlled by O_2_ supply from leaf photosynthesis, O_2_ in overlying water and transport through the porous root structure, and constant consumption in sediment [16,18,38]. The highest ROL rate and O_2_ concentration (Figure 2; Table 1 and Table 2) occurred locally around the basal root of the root system (basal root, lateral root and root tip). This was due to the shortest distance from the O_2_ source to the basal root and the lowest O_2_ consumption near the basal root [48] and its connection with O_2_-enriched overlying water for downward O_2_ diffusion [49,50]. The measured OPD range (1.30 ± 0.20–11.14 ± 2.24 mm) (Table 1) indicated that the thickness of the aerobic sediment layer increased along with the increment in illumination intensity or O_2_ concentration in water. O_2_ transport from leaf photosynthesis into overlying water and O_2_ in overlying water from aeration can diffuse across the diffusive boundary layer and oxidize the upper layer of sediment, which leads to OPD in sediment [10], while O_2_ transport from porous roots to rhizosphere sediment brings out OPD in the root system [13].

ROL capabilities differ significantly among different kinds of plants. The ROL rates of *V. spiralis* in this investigation (Table 1) are compared to other aquatic plants reported in some references [6,10,13,37,51]. The ROL rate values (13.1 ± 4.6–49.6 ± 9.5 nmol m^−2^ s^−1^, n = 10) for *V. spiralis* in this experiment were comparable to 8.80 ± 7.32–30.34 ± 17.71 nmol m^−2^ s^−1^ in the other PO research on *V. spiralis* [6] and 28.90 ± 9.69–38.66 ± 18.03 nmol m^−2^ s^−1^ for another species (*Acorus calamus*) [10], lower than 5.32–122.69 nmol m^−2^ s^−1^ for rice roots [37] and 183.33–316.67 nmol m^−2^ s^−1^ for another species (*Littorella uniflora*) [51], and higher than 4.81 ± 1.73–13.05 ± 4.96 nmol m^−2^ s^−1^ for *Phragmites australis* [13], 17.24 ± 7.20–20.06 ± 8.58 for another species (*Oryza sativa*) [10] and 10.03 ± 3.64–13.03 ± 5.37 for *V. spiralis* [10]. The difference between *V. spiralis* and the plants reported in some references is due to interspecific differences in root morphology [52,53], different environmental conditions and PO images taken in different plant growth periods [6]. The well-developed aerenchyma and cortical porosity, and the large root diameter for plants such as rice [37] and *Acorus calamus* [10], improved diffusion and transport of O_2_ towards the root. The various environmental conditions of light intensity [50] and O_2_ aeration in overlying water [38] in the abovementioned plant cultivations can also lead to ROL diversity for those plants. According to the research results in references [6,51], ROLs of 30.34 ± 17.71 nmol m^−2^ s^−1^ for the young plants of *V. spiralis* [6] and 316.67 nmol m^−2^ s^−1^ for *Littorella uniflora* [51] were significantly higher than 8.80 ± 7.32 nmol m^−2^ s^−1^ and 183.33 nmol m^−2^ s^−1^ for their old roots, respectively. In this experiment, the high light intensity and O_2_ concentration in overlying water favored O_2_ leakage from the roots of *V. spiralis* [38]. ROL-derived O_2_ release from *V. spiralis* roots diffused several mm into the sediment, which created an oxidized area around the roots (Figure 2 and Figure 4; Table 1). The increased oxidized zone is important for the survival and quick growth of *V. spiralis* in anoxic and toxic sediments [38].

### 4.2. Influence of Irradiance and Oxygen in Overlying Water on O_2_ Dynamics in the Rhizosphere

This investigation exactly detected and derived the ROL capabilities and O_2_ distribution characteristics in the rhizosphere of *V. spiralis* by a high-resolution PO method. The 2D O_2_ image, extracted 1D O_2_ profile and ROL parameters have provided clear evidence for O_2_ distribution variation along and across various root parts under four environmental conditions (Figure 2, Figure 3 and Figure 5; Table 1 and Table 2). This result was consistent with that reported in references [10,13], which suggested that light intensity, O_2_ level in overlying water and root morphology affected O_2_ leakage dynamics in root systems, resulting in the characteristics of the oxygenated area and ROL parameters (OPD, DOU and ROL rate) of oxic roots in bulk anoxic sediment.

Root-induced O_2_ leakage is the result of complex interactions between O_2_ sinks and environmental conditions [22,25]. The lowest ROL rate, O_2_ concentration in root parts and oxygenated area % in rhizosphere sediment under darkness/low O_2_ of the four environmental conditions (Figure 4; Table 1 and Table 2) were attributed to (1) the lowest O_2_ concentration in water for transfer to plant aerenchyma and (2) depletion in photosynthesis under darkness [6,10,13,49,52,53]. On the contrary, high illumination intensity enhanced the photosynthetic activity of *V. spiralis* leaves, and the high water O_2_ level engendered the transfer of sufficient oxygen in the water column through the leaf and plant aerenchyma to the root, according to the research results in references [3,37,50,54,55,56,57]. So, the highest ROL rate, oxygenated area % in the rhizosphere and O_2_ concentration in the root parts were observed under high illumination/high O_2_ (Figure 5d; Table 1 and Table 2). Though the values of oxygenated area % (Figure 4) related to the aerobic sphere in the rhizosphere under darkness/low O_2_ and darkness/high O_2_ were reduced, the aerobic sphere near the root parts was still maintained and did not disappear (Figure 5c,e). This suggested the adaptive ability of *V. spiralis* to deal with depleted photosynthetic activity, including (1) O_2_ exchange between overlying water and aboveground plant parts (leaf and stem) in darkness [6,17] and (2) internal O_2_ accumulation during photosynthesis to maintain root oxygenation in darkness [10,25].

ROL parameters (Table 1), O_2_ concentrations in three root parts (Table 2), and oxygenated area % in the rhizosphere (Figure 4) under darkness/high O_2_ were significantly higher than under darkness/low O_2_. O_2_ stored in roots and especially O_2_ transfer from overlying water supplemented the O_2_ level in the rhizosphere under darkness conditions, according to the research results in references [10,15]. Similar to *V. spiralis*, the roots of *Zostera marina* [18] and *Phragmites australis* [13] can also maintain O_2_ leakage in the rhizosphere without photosynthesis when the overlying water remains aerobic.

### 4.3. Implications of the O_2_ Gradient Across the Root Surface and the Longitudinal O_2_ Distribution Along the Root Axis 

The average O_2_ maximum in the line transecting each kind of root followed the sequence basal root > lateral root > root tip under various environmental conditions (Table 3). This was due to the different distances from the O_2_ source in the aboveground part of *V. spiralis* or overlying water to the different positions of the root parts mentioned above [16,48,57]. Exponential O_2_ depletion from the root center (maximum O_2_) to the fringe of the rhizosphere (minimum O_2_) was found in each cross-sectional profile (a, b, c or d) across one typical root (Figure 6A,B). It was controlled by O_2_ secreted from the root surface and enhanced O_2_ consumption from the root surface to bulk sediment, for example, microbial respiration, chemical oxidation and organic decomposition [16,18,57]. A similar O_2_ distribution gradient for a typical plant root has also been found in *Cymodocea rotundata* and *Zostera marina*, as reported in references [5,12].

O_2_ was secreted from the root surface of *V. spiralis* and diffused into the rhizosphere sediment. This resulted in an aerobic area around lines (a–d) across the root axis (Figure 6A) with a width of 2.22 ± 0.10–3.20 ± 0.14 mm (n = 7) (Figure 6B). The average O_2_ concentration for each radial line (a, b, c or d) continuously increased from 91.5 ± 5.9 μmol L^−1^ (n = 7) on line (a) at the root tip to 111.3 ± 6.2 μmol L^−1^ (n = 7) on line (d) at the top of the root (Figure 6B). The maximum O_2_ (147.4 ± 3.7 μmol L^−1^, n = 7) on line (d) at the top of the root decreased to 131.2 ± 2.4 μmol L^−1^ (n = 7) on line (a) at the root tip (Figure 6C and Table 3). Moreover, the O_2_ concentration gradient on line (e) also demonstrated a depletion tendency from the top of the root to the root tip along the root axis (Figure 6C and Table 3). The distance from the aboveground part of *V. spiralis* to the root tip was longer than that of the top of this root, which caused the O_2_ concentration at the top of the root to be larger than that at the root tip [16,57]. This has also been found for some plants, such as *Lobelia dortmanna* [58] and *Juncus effusus* [59], which exhibit significant O_2_ release along the root axis. However, other plants, such as *Spartina anglica* [3] and *Potamogeton crispus* [17], demonstrate the opposite ROL pattern, and their below-ground O_2_ leakage is restricted to the root tip.

## 5. Conclusions

This research has visualized the complex dynamics of O_2_ in the rhizosphere of *V. spiralis* in response to light/dark transitions and O_2_ levels in overlying water using a high-resolution planar O_2_ optode. The spatial heterogeneity in rhizosphere O_2_ distribution and O_2_ dynamics in different root parts under environmental fluctuations are revealed by 2D images of O_2_ distribution, the derived 1D vertical O_2_ profile and ROL parameters. The O_2_ dynamics in the rhizosphere are highly changeable and depend on light irradiance and O_2_ concentration in overlying water. The O_2_ concentration distribution and ROL rate for different root parts and oxygenated area % in rhizosphere sediment decrease significantly, along with the depletion of illumination intensity or O_2_ concentration in overlying water. Photosynthesis-derived O_2_ production under illumination, the O_2_ gradient between overlying water and sediment, and O_2_ transfer to plant aerenchyma and roots influence O_2_ distribution patterns in the rhizosphere, ROL rates and oxygenated area %. The O_2_ level in water has a more distinct effect on the ROL of *V. spiralis* than the illumination–darkness transition. The ROL rate and O_2_ concentration for three root parts follow the sequence basal root > lateral root > root tip, which is due to the different distances from the O_2_ source in the aboveground part of *V. spiralis* or overlying water to the root part (basal root, lateral root or root tip). O_2_ consumption in rhizosphere sediment and O_2_ secretion from the root surface lead to a maximum O_2_ concentration in the root center and a minimum in the rhizosphere fringe.

Altogether, O_2_ leakage from roots to the rhizosphere is influenced by light–darkness and the exchange between water O_2_ and plant tissues (leaf and stem). The various ROL characteristics and O_2_ dynamics in different root parts and the rhizosphere reflect the resilience and ecological adaptability of *V. spiralis* in O_2_-deficient sediment. The planar O_2_ optode has been proven to be an excellent 2D visualization tool for highly dynamic ROL in heterogeneous oxidation-reduction rhizosphere sediment. Further research should be devoted to investigating the effects of temperature and altitude of water bodies on ROL, the uptake, transfer and transformation of nutrients in response to ROL in rhizosphere sediment, and the phytoremediation mechanism of macrophyte roots.

## Figures and Tables

**Table 1:** The average values of the width of the oxygenated zone at different surface positions (L), root diameter (A), O_2_ penetration depth (OPD), diffusive O_2_ uptake (DOU), volume-specific O_2_ consumption (*R_SWI_*) and radial O_2_ loss rate (ROL rate). Note: The values are reported as mean ± SD (n = 7).

Environmental Condition	Rhizobox	OPD	DOU	*R_SWI_*	ROL Rate	A	L
		(mm)	(nmol m^−2^ s^−1^)	(μmol m^−3^ s^−1^)	(nmol m^−2^ s^−1^)	(mm)	(mm)
High illumination/low O_2_	SWI	8.04 ± 0.98	14.3 ± 3.0	1.8 ± 0.4			
	Basal root	2.16 ± 0.32	−	−	32.2 ± 8.3	0.24 ± 0.02	3.02 ± 0.16
	Lateral root	1.94 ± 0.21	−	−	29.3 ± 7.3	0.20 ± 0.01	2.64 ± 0.14
	Root tip	1.76 ± 0.32	−	−	22.0 ± 6.2	0.16 ± 0.01	2.04 ± 0.12
Darkness/low O_2_	SWI	6.45 ± 0.89	10.8 ± 1.6	1.7 ± 0.5			
	Basal root	1.85 ± 0.29	−	−	20.6 ± 5.1	0.24 ± 0.02	2.45 ± 0.14
	Lateral root	1.65 ± 0.25	−	−	14.1 ± 4.1	0.20 ± 0.01	1.83 ± 0.11
	Root tip	1.30 ± 0.20	−	−	13.1 ± 4.6	0.16 ± 0.01	1.59 ± 0.14
High illumination/high O_2_	SWI	11.14 ± 2.24	13.8 ± 2.0	1.2 ± 0.2			
	Basal root	2.83 ± 0.38	−	−	49.6 ± 9.5	0.24 ± 0.02	4.71 ± 0.16
	Lateral root	2.22 ± 0.36	−	−	36.6 ± 8.3	0.20 ± 0.01	3.68 ± 0.19
	Root tip	1.94 ± 0.27	−	−	28.8 ± 6.4	0.16 ± 0.01	2.92 ± 0.16
Darkness/high O_2_	SWI	8.95 ± 1.84	11.9 ± 1.6	1.3 ± 0.3			
	Basal root	2.31 ± 0.60	−	−	39.2 ± 7.5	0.24 ± 0.02	3.99 ± 0.15
	Lateral root	1.98 ± 0.28	−	−	36.2 ± 6.5	0.20 ± 0.01	3.51 ± 0.14
	Root tip	1.51 ± 0.22	−	−	22.7 ± 6.2	0.16 ± 0.01	2.58 ± 0.14

**Table 2:** The maximum O_2_ concentration on the line transecting the basal root, lateral root or root tip under high illumination/low O_2_, darkness/low O_2_, high illumination/high O_2_ or darkness/high O_2_ and the average O_2_ concentration. Note: The average [1] is the averaged O_2_ concentration for the basal root, lateral root or root tip for all four environmental conditions; the average [2] is the averaged O_2_ concentration for the basal root, lateral root or root tip under each environmental condition. Note: The values are reported as mean ± SD (n = 7).

Root Classification	The Maximum O_2_ Concentration in Each Root Line				The Average [1]
	High Illumination/Low O_2_	Darkness/Low O_2_	High Illumination/High O_2_	Darkness/High O_2_	(μmol L^−1^)
	(μmol L^−1^)	(μmol L^−1^)	(μmol L^−1^)	(μmol L^−1^)	
**Base root**					
1	160.0 ± 2.4	146.9 ± 1.9	172.1 ± 3.4	172.1 ± 4.6	162.8 ± 3.2
**Root tip**					
2	121.0 ± 1.9	97.4 ± 1.4	124.9 ± 2.2	111.4 ± 1.2	
3	121.2 ± 2.3	63.4 ± 1.1	114.9 ± 1.6	100.6 ± 1.2	
4	142.1 ± 2.2	107.4 ± 1.5	143.2 ± 2.8	114.8 ± 2.4	
X			141.4 ± 2.3	139.9 ± 2.4	
**The average [2]**					
**(μmol L^−1^)**	128.1 ± 2.3	89.4 ± 1.2	131.1 ± 2.9	116.7 ± 2.2	120.3 ± 2.3
**Lateral root**					
5	146.9 ± 3.0	101.7 ± 0.9	146.9 ± 3.2	121.6 ± 2.4	
6	171.8 ± 3.2	121.7 ± 2.4	171.8 ± 3.4	146.9 ± 3.2	
7	127.0 ± 2.3	100.8 ± 2.2	130.2 ± 2.8	125.5 ± 2.4	
8	119.1 ± 2.2	94.7 ± 1.6	165.4 ± 3.0	150.4 ± 3.2	
**The average [2]**					
**(μmol L^−1^)**	141.2 ± 3.4	104.7 ± 2.0	153.6 ± 3.2	136.1 ± 2.8	133.9 ± 2.4

**Table 3:** The sequence of the average O_2_ concentrations in the basal root, lateral root and root tip under four environmental conditions and the sequence of O_2_ concentrations at different locations on lines (a–e) in one typical root under high illumination/low O_2_. Note: The values are reported as mean ± SD (n = 7). Notice: The arrows and equals signs are used for numerical magnitude comparison.

Root Parts	The Average O_2_ Concentration (μmol L^−1^)								
	All Four Conditions	High Illumination/High O_2_		Darkness/High O_2_		High Illumination/Low O_2_		Darkness/Low O_2_	
Basal root	162.8 ± 3.2	172.1 ± 3.4	=	172.1 ± 4.6	←	160.0 ± 2.4	←	146.9 ± 1.9	
	↓	↓		↓		↓		↓	
Lateral root	133.9 ± 2.4	153.6 ± 3.2	←	136.1 ± 2.8	→	141.2 ± 3.4	←	104.7 ± 2.0	
	↓	↓		↓		↓		↓	
Root tip	120.3 ± 2.3	131.1 ± 2.9	←	116.7 ± 2.2	→	128.1 ± 2.3	←	89.4 ± 1.2	
Radial O_2_ lines across root/O_2_ concentration (μmol L^−1^)					Longitudinal O_2_ profile/O_2_ concentration (μmol L^−1^)				
Four lines (a–d)		root center		rhizosphere fringe	O_2_ profile		top of root axis		near root tip
a		131.2 ± 2.4	→	47.2 ± 1.4	e		160.9 ± 3.2	→	85.3 ± 2.4
b		141.2 ± 2.8	→	50.3 ± 1.6					
c		136.8 ± 3.2	→	63.0 ± 2.0					
d		147.4 ± 3.7	→	75.9 ± 2.2					

## Data Availability

The original contributions presented in this study are included in the article/Supplementary Materials. Further inquiries can be directed to the corresponding author.

## Supplementary Materials

The following supporting information can be downloaded at https://www.mdpi.com/article/10.3390/plants15131935/s1, Figure S1: The location of one sampling site at Qing River in Beijing (China). The map is derived by Google Earth Pro (Version 7.3.7); Figure S2: (A): The locations for seven O_2_ vertical profiles in each image (Such as Figure 2a) for derivation of *∂C*/*∂Z* and DOU or data analysis in Section 2.4 in text; (B): The locations for seven O_2_ vertical profiles in rhizosphere sediment in each image (such as Figure 2a) for data analysis in Section 2.4 in text.

## Abbreviations

The following abbreviations are used in this manuscript:

*V. spiralis*	*Vallisneria spiralis*
ROL	radial oxygen loss
2D	two-dimensional
PO	planar optode
TP	total phosphorus P
TN	total nitrogen
TFe	total iron
CMOS	complementary metal oxide semiconductor
LED	light-emitting diode
OPD	O_2_ penetration depth
DOU	diffusive O_2_ uptake
*R_SWI_*	volume-specific O_2_ consumption
