# Supplementary material for: Oxygen Dynamics in the Rhizosphere of Vallisneria spiralis Characterized by a Fluorescent Planar Optode
Source: Plants (Basel). 2026 Jun 23;15(13):1935. doi: 10.3390/plants15131935 (PMC13364264; doi:10.3390/plants15131935)
Supplement: Supplementary file 1 [file plants-15-01935-s001.zip › plants-4311080-supplementary.pdf]

Supplementary Material for

Oxygen Dynamics in the Rhizosphere of *Vallisneria spiralis* Characterized by Fluorescent Planar Optode

Jingwei Tan [1, 2, 3], Zhihao Wu [1, 2, 3], Xiaosong Yang [1, 2, 3], Weidong Jin [1, 2, 3] *, Yiming Zhao[1, 2, 3], Qing Cai [1, 2, 3]

1. National Engineering Laboratory for Lake Pollution Control and Ecological Restoration, Institute of Lake Environment, Chinese Research Academy of Environmental Sciences (CRAES), Beijing 100012, China.

2. State Environmental Protection Key Laboratory for Lake Pollution Control, Institute of Lake Environment, Chinese Research Academy of Environmental Sciences (CRAES), Beijing, 100012, China.

3. State Key Laboratory of Environmental Criteria and Risk Assessment, Chinese Research Academy of Environmental Sciences (CRAES), Beijing, 100012, China.

*Correspondence: kindong.04@163.com (W.J.)

The information

Figure: 2

SM A and B

Figure S1 The location of one sampling site at Qing River in Beijing (China). The map is derived by Google Earth Pro (Version 7.3.7).

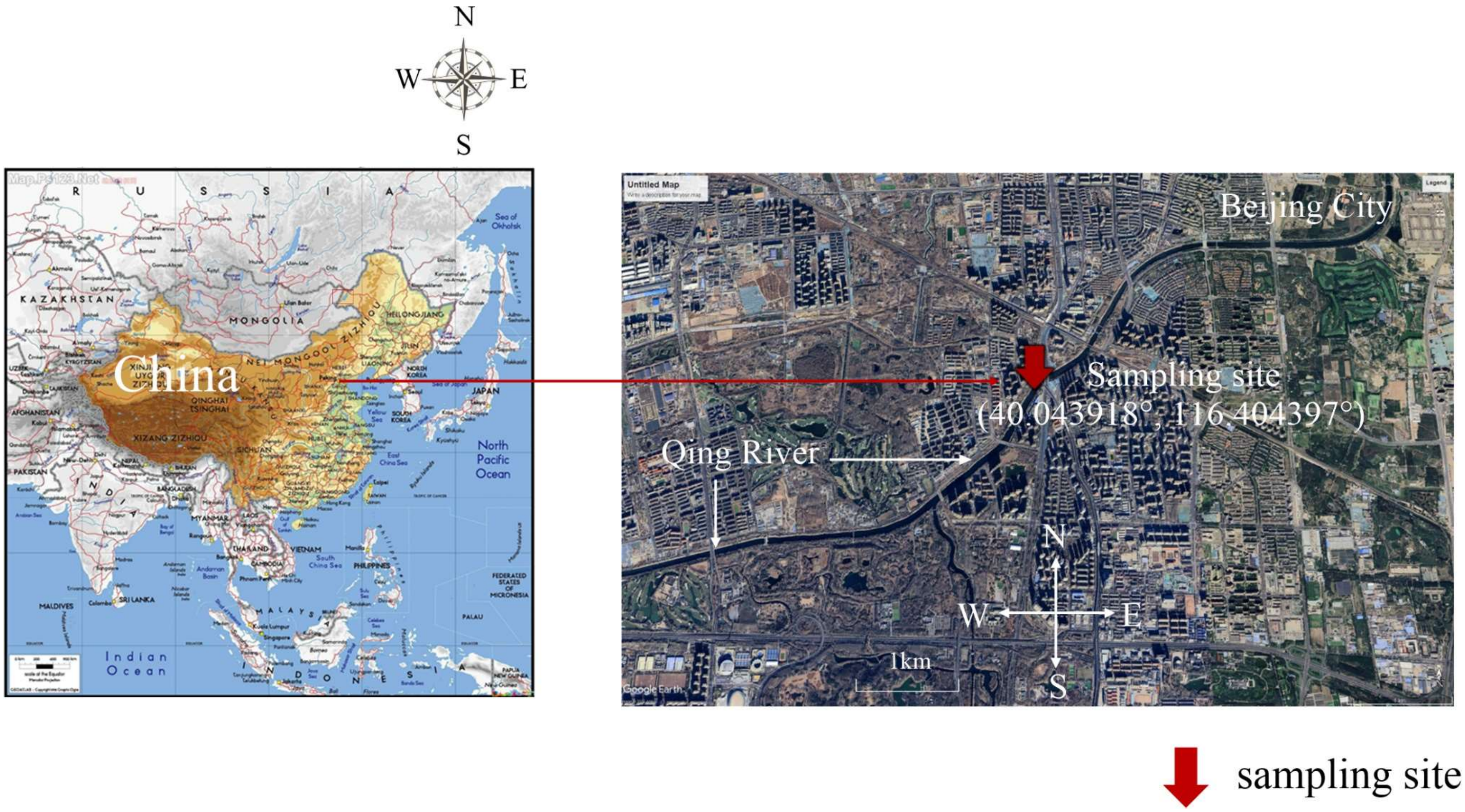

Figure S2: A: The locations for seven O_2 vertical profiles in each image (Such as Figure 2 a) for derivation of $\frac{\partial C}{\partial Z}$ and DOU or data analysis in Sect. 2.4 in text; B: The locations for seven O_2 vertical profiles in rhizosphere sediment in each image (such as Figure 2a) for data analysis in Sect. 2.4 in text.

(A)

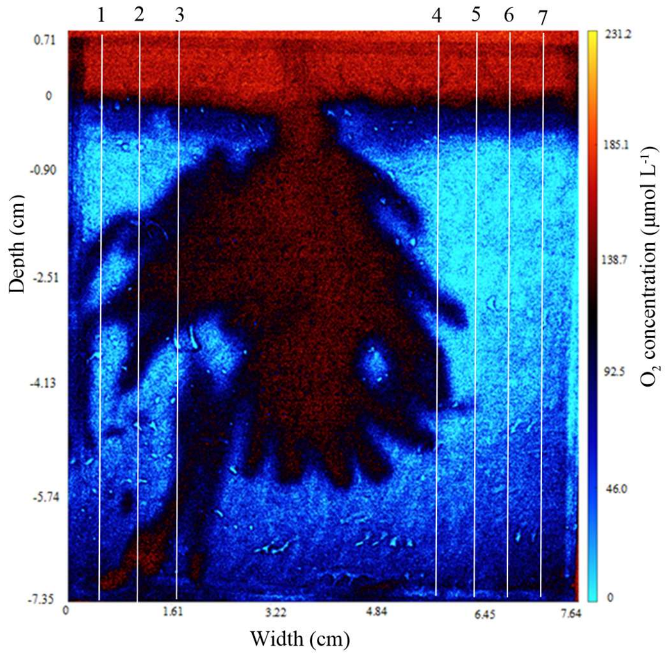

(B)

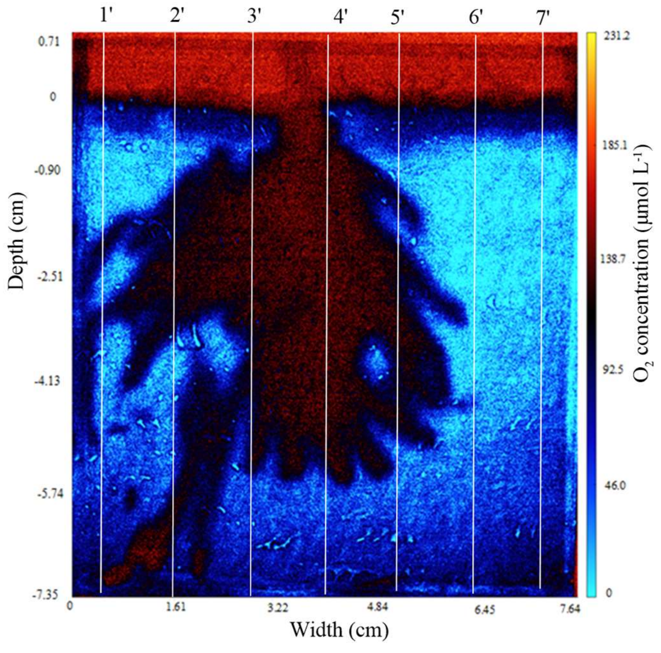

SM A

The cultivation for *V. spiralis*, the method for controlling environmental conditions and the procedures for root system and sediment after PO measurement

The rectangular rhizobox with one detachable front window (Fig.1) made of Perspex and the inner size is height (20 cm)×length (10 cm)×width (7 cm). The detachable front window was transparent, and the other three walls and the base were lightproof. One seedling of *V. spiralis* was chosen for one rhizobox and the length and weight for the seedling was 10±0.4 cm and 3.2±0.2 g. The length of the root was only 2.0 cm. The seedling was planted adjacent to the front window of rhizobox. The front window was covered by a black plastic sheeting; then the rhizobox with plant was put into one experimental flume with illumination lamp, aeration equipment (high pressure vessels with air or 30 %-N_2), water return equipment, and water. The rhizobox was kept at an angle of 30° to ensure that the roots developed alongside the front window. The total P (TP), total N (TN) and total Fe (TFe) in sediment were 984, 2889 and 15854 mg kg^{-1}, respectively. The overlying water with the volume of 200 ml and TP (30.4 μg L^{-1}) and TN (74.1μg L^{-1}) was pooled into rhizobox; the sediment (0.8 kg) were mixed with water. The plant was cultivated in experimental flume for 12 days with photoperiod of a 12:12 h illumination-darkness cycle (light intensity of 0 or 150 μmol photons m^{-2} s^{-1}), O_2 concentration in water in the experimental flume (175 μmol L^{-1}) and temperature of 20 °C. Without aeration in water in experimental flume, O_2 in water (175 μmol L^{-1}) in rhizobox is measured using a multi-parameter water quality analyzer (HQ 4300, Hach Company, USA). An illumination lamp was used to control the irradiation intensity of

150 μmol photons m^{-2} s^{-1} over rhizobox, which was measured using a light quantum meter (3415 FSE, Spectrum Technologies Inc., USA).

After 12 d cultivation, the plant's growth reached a steady-state phase and the experiment for in-situ O_2 measurement can be conducted. The rhizobox was taken out from the experimental flume (Fig.1) and the detachable front window was taken off. One O_2 sensing foil (length: 8 cm×width: 8 cm) was attached to the detachable front window. The detachable front window attached with the O_2 sensing film was fixed on rhizobox. A black plastic sheeting was attached to the outside of this detachable front window. The rhizobox was placed at an angle of 30° in the experimental flume to ensure the development of root along the front window. The cultivation time was 4 h under one environmental condition. The roots can develop along the front window of rhizobox. In order to investigate the effect of O_2 concentrations in water in experimental flume or illumination on O_2 dynamics in rhizosphere, the macrophyte was exposed to two O_2 concentrations (141 or 280 μmol L^{-1}) in water, and 0 or 200 μmol photons m^{-2} s^{-1} of light intensity.

An aeration head connected with a gas supply system was used to control O_2 in water in experimental flume. The gas supply system can supply (1) pure O_2 or (2) mixed gas (air: nitrogen=6:4) with aeration rate of 1.4 L min^{-1} to sustain 280 or 141 μmol L^{-1} O_2 concentration in water in experimental flume, which is measured using a multi-parameter water quality analyzer (HQ 4300, Hach Company, USA). When O_2 in water is maintained at 141 or 280 μmol L^{-1} for 1 h with RSD $\leq$ 5 %, O_2 in water can be confirmed as stability.

The irradiation with 0 or 200 μmol photons m^{-2} s^{-1} was controlled by a black plastic sheeting for packing the experimental flume, an illumination lamp and a light quantum meter (3415 FSE, Spectrum Technologies Inc., USA). When light intensity is maintained at 0 or 200 μmol photons m^{-2} s^{-1} for 1 h with RSD ≤ 5 %, the irradiation intensity can be confirmed as stability.

The temperature of water in experimental flume and rhizobox was 20 ºC. TP and TN in overlying water were 20.2 and 65.4 μg L^{-1}, respectively. The sequence of four conditions occurring in rhizobox is demonstrated as: illumination (200 μmol photons m^{-2} s^{-1})/O_2 (141 μmol L^{-1}) in overlying water; darkness (0 μmol photons m^{-2} s^{-1})/O_2 (141 μmol L^{-1}); illumination (200 μmol photons m^{-2} s^{-1})/O_2 (280 μmol L^{-1}) and darkness (0 μmol photons m^{-2} s^{-1})/O_2 (280 μmol L^{-1}). Four conditions in rhizobox in the experimental flume appeared in sequence during 16 h. Four environmental conditions mentioned above can also be demonstrated as: high illumination/low O_2; darkness/low O_2; high illumination/high O_2 and darkness/high O_2. The rhizobox was put into water in experimental flume for stabilization under each environmental condition for 4 h. After 4 h, the rhizobox was transferred to the enclosed PO device for O_2 measurement in rhizosphere sediment. The environmental condition was changed to the next one and the rhizobox was put into water in experimental flume for the next 4 h stabilization.

After PO measurements under four environmental conditions, the detachable front window for rhizobox was removed and a digital camera (Canon EOS 600D, Japan) was used to take photos of the rhizosphere. The root and sediment were removed from

rhizobox carefully. The plant was washed using deionized water (Milli-Q Element 18 MU) and after that, the root system was soaked in the deionized water to remove the sediment adhered to root. Then, the diameters for basal root, lateral root or root tip (n=7) were measured using a digital vernier caliper (543-781, Mitutoyo Company, Japan) with a resolution of 0.01 mm and the average root diameter (n=7) was derived. Root length was also measured using the same method.

The sediment layer from 0 to -8.0 cm was sliced at a vertical resolution of 1.0 cm. Each wet sediment layer was weighted. Then it was dried at 105 °C for dry weight. The particle concentration in sediment (Pc) (g cm^{-3}) can be calculated through SM Eq.(1):

$$\mathrm{Pc}=m/v \qquad \text{SM Eq.(1)}$$

where, m is the total mass of the sediment particles (g); v (cm^3) is the volume of porewater in a given volume of total sediment.

The sediment porosity (ϕ) can be derived by SM Eq.(2) in reference [45]:

$$\phi = dp/(P_c+dp) \quad \text{SM Eq.(2)}$$

where, dp (g cm^{-3}) is the density of sediment particle, which is usually assumed to be 2.65 g cm^{-3}.

SM B

Principle, fabrication and calibration of O_2 planar optode

The imaging principle of O_2 PO measurement, is based on the dynamic quenching of a fluorophore in the presence of O_2 [3]. O_2 sensing film can be fabricated by coating the “cocktail” includes an analyte-sensitive indicator dye, an analyte-insensitive reference dye and an analyte-permeable polymer on a transparent dust-free

polyethylene terephthalate [22]. The analyte concentrations are quantified by the ratio of indicator and reference signal through calibration. In this study, the O_2 sensing film was prepared by two steps: firstly, the sensor "cocktail" was prepared by dissolving 20 mg reference fluorophore coumarin C545 (C545T) and 10 mg Platinum (II) 5,10,15,20-tetrakis-(2,3,4,5,6-pentafluorphenyl) porphyrin (PtTFPP), 500 mg polystyrene in 10 mL toluene. The "cocktail" was then gently stirred and sprayed on a transparent dust-free polyethylene terephthalate using ultrasonic spray equipment. Secondly, a thin layer of silicone rubber (Dow Corning 3140) was coated on top of the dry sensing layer as a protective layer, and the silicone layer was further cured for 24 h.

The calibration process, i.e. a two-point calibration procedure, was performed using deionized water with varying oxygen concentrations at 25 ^{O}C. The oxygen-free water (0%-O_2) was prepared using sodium dithionite. The O_2 bubbled overlying water was used for 100 %-O_2 water. The O_2 concentration in water was measured by Pen-type meter (NPT-DO 601, Shanghai Nobo Environmental Technology Co., Ltd., China). The fluorescence intensity ratio of Red and Green was applied for measuring O_2 concentration. A modified Stern–Volmer equation was used to measure the O_2 concentration according to the fluorescence intensity ratio as the equation 3 (SM) in reference [6]:

$$\frac{R}{R_0} = \alpha + (1-\alpha)\frac{1}{1+K_{SV}C} \quad \text{(SM Eq.3)}$$

Where R and R_0 are the fluorescence intensity ratio (Red/Green) in at an O_2 concentration of C (%, air saturation) and C_0 (0 %, air saturation), respectively., C is the O_2 concentration, K_{sv} is the Stern-Volmer quenching constant, α is the non-

quenchable fraction of the fluorescence signal, and it is temperature independent. In addition, the calibration curves in different temperatures were made by calibrating O_2 planar optode in different O_2 deionized water under the corresponding temperature. The imaging system PO2100 (https://global-easysensor.com/) was used to obtain the color and fluorescent images. This system comprises a complementary metal-oxide-semiconductor (CMOS) camera equipped with a macro lens and a 460-nm long-pass filter, and a computer to control both the light sources (390-400 nm) and the camera.

References

[3] Koop-Jakobsen K, Wenzhofer F (2015) The dynamics of plant-mediated sediment oxygenation in Spartina anglica rhizospheres-a planar optode study. Estuar Coast 38 (3): 951–963.

[6] Han C, Ren J H, Tang H, Xua D, Xie X C (2016) Quantitative imaging of radial oxygen loss from Valisneria spiralis roots with a fluorescent planar optode. Sci Total Environ 569: 1232-1240.

[22] Li C, Ding S, Yang L, Zhu Q, Chen M, Tsang D C W, Cai G, Feng C, Wang Y, Zhang C (2019) Planar optode: a two-dimensional imaging technique for studying spatial-temporal dynamics of solutes in sediment and soil. Earth Sci Rev 197: 102916-102934.

[45] Zhang H, Lombi E, Smolders E, Mcgrath S (2004) Kinetics of Zn release in soils and prediction of Zn concentration in plants using diffusive gradients in thin films. Environ Sci Technol 38: 3608-3613.
